# Comparative genomics of 274 *Vibrio cholerae* genomes reveals mobile functions structuring three niche dimensions

**DOI:** 10.1186/1471-2164-15-654

**Published:** 2014-08-05

**Authors:** Bas E Dutilh, Cristiane C Thompson, Ana CP Vicente, Michel A Marin, Clarence Lee, Genivaldo GZ Silva, Robert Schmieder, Bruno GN Andrade, Luciane Chimetto, Daniel Cuevas, Daniel R Garza, Iruka N Okeke, Aaron Oladipo Aboderin, Jessica Spangler, Tristen Ross, Elizabeth A Dinsdale, Fabiano L Thompson, Timothy T Harkins, Robert A Edwards

**Affiliations:** Department of Biology, San Diego State University, San Diego, CA USA; Department of Computer Science, San Diego State University, San Diego, CA USA; Centre for Molecular and Biomolecular Informatics, Radboud Institute for Molecular Life Sciences, Radboud University Medical Centre, Nijmegen, The Netherlands; Department of Marine Biology, Institute of Biology, Federal University of Rio de Janeiro, Rio de Janeiro, Brazil; Laboratory of Molecular Genetics of Microorganisms, Oswaldo Cruz Institute, FIOCRUZ, Rio de Janeiro, Brazil; Computational Science Research Center, San Diego State University, San Diego, CA USA; Advanced Applications Group, Life Technologies, Inc, Beverly, MA USA; Department of Biology, Haverford College, Haverford, PA USA; Department of Medical Microbiology & Parasitology, College of Health Sciences, Obafemi Awolowo University, Ile-Ife, Nigeria; Mathematics and Computer Science Division, Argonne National Laboratory, Argonne, IL USA

**Keywords:** Functional genomics, Mobile elements, Phages, Niche adaptation, Vibrio, Genome evolution, Genotype-phenotype association, Random forest

## Abstract

**Background:**

*Vibrio cholerae* is a globally dispersed pathogen that has evolved with humans for centuries, but also includes non-pathogenic environmental strains. Here, we identify the genomic variability underlying this remarkable persistence across the three major niche dimensions space, time, and habitat.

**Results:**

Taking an innovative approach of genome-wide association applicable to microbial genomes (GWAS-M), we classify 274 complete *V. cholerae* genomes by niche, including 39 newly sequenced for this study with the Ion Torrent DNA-sequencing platform. Niche metadata were collected for each strain and analyzed together with comprehensive annotations of genetic and genomic attributes, including point mutations (single-nucleotide polymorphisms, SNPs), protein families, functions and prophages.

**Conclusions:**

Our analysis revealed that genomic variations, in particular mobile functions including phages, prophages, transposable elements, and plasmids underlie the metadata structuring in each of the three niche dimensions. This underscores the role of phages and mobile elements as the most rapidly evolving elements in bacterial genomes, creating local endemicity (space), leading to temporal divergence (time), and allowing the invasion of new habitats. Together, we take a data-driven approach for comparative functional genomics that exploits high-volume genome sequencing and annotation, in conjunction with novel statistical and machine learning analyses to identify connections between genotype and phenotype on a genome-wide scale.

**Electronic supplementary material:**

The online version of this article (doi:10.1186/1471-2164-15-654) contains supplementary material, which is available to authorized users.

## Background

Species exist in a multi-dimensional niche space with dimensions representing their environmental habitat, their geographical location, and their presence in time. Within this space, each species occupies a volume with a specific shape and size. Some species may occur as relatively tight clusters, if they have a strict habitat preference (habitat), are incapable of distant migration (space), and quickly go extinct (time). Other species persist, forming large structured networks in niche space. One example of the latter is *Vibrio cholerae*, the causative agent of cholera that has persisted globally for centuries in clinical as well as environmental habitats.

Here, we ask the question which elements of the *V. cholerae* genome reflect its structured occurrence in each of the three niche dimensions. Glimpses of an answer can be found in the literature. For example, a recent phylogeographic analysis distinguished two gene pools [1]: the vertically inherited core genome and horizontally acquired mobile or mobilizable elements. These mobile elements sweep through local subpopulations of environmental *Vibrio* species that occupy the same geographical niche, leading to geographic structuring [2]. Temporal structuring of the *V. cholerae* genome was shown in a large-scale sequencing effort of *V. cholerae* strains from the sixth (1899-1923) and seventh (1961 onwards) cholera pandemics [3], that identified genomic elements characterizing the different temporal waves of the pandemic. Finally, habitat occupancy is mainly regulated by the presence of two phage-encoded factors. Clinical, epidemic *V. cholerae* strains differ from non-pathogenic environmental ones depending on the presence of the toxin co-regulated pilus (TCP) that allows the bacterium to colonize the gut and form protective aggregates; and cholera toxin (CTX), encoded by the phage CTXφ, which uses the TCP to attach to the bacterium [4].

We have recently outlined an approach for genome-wide association of genotypes to phenotypes (GWAS for microbes or GWAS-M) that is capable of exploiting large-scale draft genome sequencing [5]. Here, we employ this approach for the data-driven discovery of genomic elements that structure *V. cholerae* across the niche dimensions space, time, and habitat. Our approach classifies the bacterial strains by phenotype by using Random Forest (RF) machine learning [6, 7], identifying which genomic elements are most important for the classification. While consistently annotated phenotypes are often lacking for sequenced bacterial strains [5], niche metadata are readily available for most publically available genome sequences, allowing us to include 235 published complete and draft genomes in our analysis. Moreover, we sequenced 39 additional genomes using the new Ion Torrent Personal Genome Machine (PGM) benchtop sequencer [8] to obtain a more complete sampling of the niche dimensions. We identify how the genomic landscape of *V. cholerae*, consisting of genotypic variables including SNPs, protein families, functions, and phages varies across the major niche dimensions time, space, and habitat.

## Results and discussion

### *Vibrio cholerae*genomes across niche dimensions

**Figure 1:**
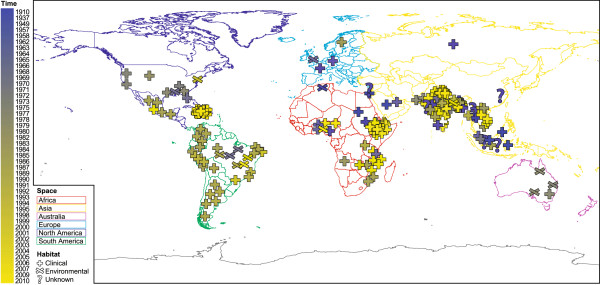
**Overview of the** ***V. cholerae*** **strains analyzed in this study.** Approximate geographical origin, isolation source and date of 274 *V. cholerae* strains. See Additional file 1 for details.

Using a total of 274 draft genome sequences, we set out to identify genomic attributes that render *V. cholerae* persistent across the niche dimensions of time, space, and habitat. We included 31 *V. cholerae* genomes present in the public genome database SEED [9], as well as 139 [3], 40 [10], 24 [11] and one [12] additional *V. cholerae* genomes that were recently sequenced. To complement these and provide a more balanced sampling of the niche dimensions, we selected an additional 39 *V. cholerae* strains for Ion Torrent sequencing from the Bacteria Culture Collection of Environment and Health at the Oswaldo Cruz Foundation (FIOCRUZ, Brazil). Together, these strains provide a good cross-section of environmental and clinical sources (habitat), different geographical regions (space), and sampling dates (time; see Figure 1 and Additional file 1).

**Figure 2:**
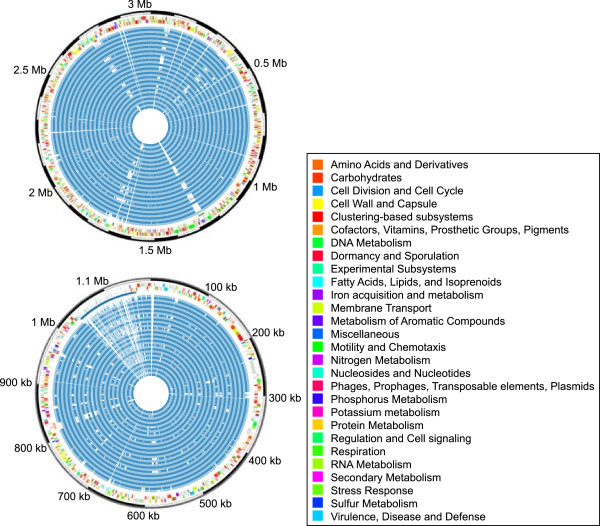
***V. cholerae*** **genome plot.** Circos plots [14] of the two major *V. cholerae* R-18308 scaffolds representing chromosome 1 (top) and chromosome 2 (bottom). From the outer circle inwards: scale; ORFs per strand (colored by functional category, see legend); prophages (orange: small defective prophage; red: CTX; green: PP1; blue: superintegron, SI); and read mappings of the 38 other sequenced genomes (blue, from the middle circle outwards: R-18246, R-18273, R-18303, R-18304, R-18316, R-18317, R-18327, R-18338, R-18348, VC08, VC1005, VC102, VC111, VC120, VC14, VC150, VC172, VC179, VC200, VC201, VC21, VC214, VC22, VC307, VC311, VC33, VC341, VC434, VC46, VC500, VC504, VC75, VC77, VC83, VC833, VC91, VC95, VC998).

The Ion Torrent platform [8] was used to sequence the strains to an estimated 94.6 ± 4.8% coverage (Additional file 2), satisfactory for the accepted standard of high quality draft genomes [13]. *V. cholerae* is endemic in Southeast Asia and most of the strains with completely sequenced genomes originated there (135 isolates). *V. cholerae* strain R-18308, an El Tor strain isolated in Asia (India 1973) and a representative of the Asiatic seventh pandemic, was sequenced very deeply (~841-fold) using a combination of shotgun and long mate pair techniques. The genome was assembled into two major scaffolds, each representing one of the two chromosomes. Figure 2 displays the two R-18308 chromosomes and their annotation, as well as the mapping of the remaining 38 genomes sequenced in this study.

### Breadth of the strains based on genome-wide marker SNPs

**Figure 3:**
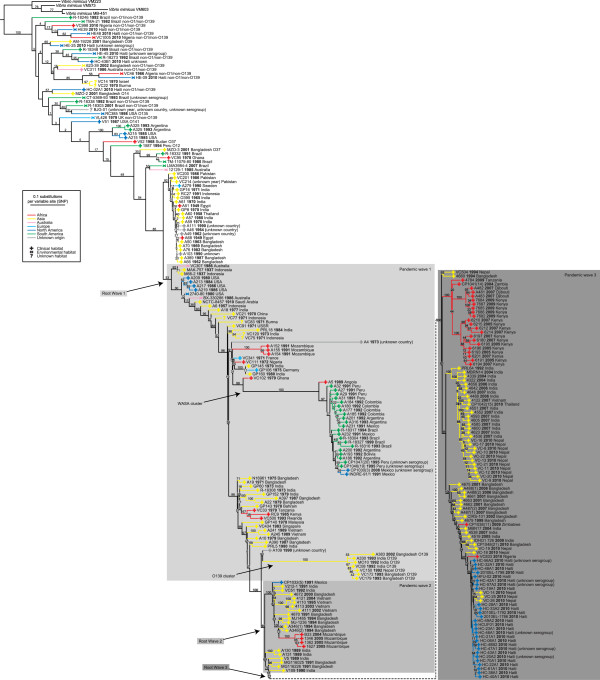
**Phylogenomic tree of** ***V. cholerae*** **genomes.** A phylogenomic tree based on genome-wide marker SNPs illustrates the breadth of 274 *V. cholerae* genomes included in this study. Four complete *V. mimicus* genomes were included as an outgroup. Branch lengths indicate the number of substitutions per SNP site. Several clusters mentioned in the text are shown. Branches are colored by the continent where the strains were isolated. The isolation source (habitat) of the strains is indicated. All strains belong to the O1 serogroup unless mentioned otherwise. Note that the transcontinental transmission event from South America to Southeast Asia (labeled “D” in reference [3], see Figure one and Supplementary Figure S3 therein) was not confirmed, and we suspect this is due to switching of the labels between strain A390 (Bangladesh 1987) and strain A316 (Argentina 1993) in that article as the positions of those strains are switched in our phylogeny.

To illustrate the breadth of the sampled strains, we mapped 1,970 previously identified marker SNPs from the sixth and seventh pandemic [3] to our genomes. A SNP-based phylogenomic tree (Figure 3) confirms the three waves of the seventh pandemic described previously [3]. Moreover, several clusters reflect local epidemics, as shown by isolates from the same geographical area and time clustering together in the tree. Examples include strains isolated in Mozambique from 1991, strains isolated in Africa from 2004 to 2009 and strains isolated in Bangladesh and Vietnam from 1995 to 2004. In 1992, a new serogroup of *V. cholerae*, O139, was identified as the cause of epidemic cholera in Bangladesh and India [15]. The O139 serogroup was genetically derived from the O1 El Tor pandemic strain after changing its antigenic structure [16, 17]. However, we confirm here that the O139 strains evolved independently from the El Tor pandemic strains, as evidenced by the tight *V. cholerae* O139 cluster. The cluster of genomes containing the West Africa-South America (WASA) phage is supported by 100% of the bootstrap iterations. Interestingly, another genome from Africa (VC102, Ghana 1979) is at the root of the WASA cluster, providing additional support for the relation of South American and West African strains as shown previously [3]. Our tree also shows that our strain VC833, isolated in September/October 2010 in Nigeria is closely related to the strains from the cholera outbreak in Haiti and Nepal of that year (Figure 3). In conjunction with previous observations [3, 11, 18], this suggests the existence of a lineage causing recent cholera outbreaks in Asia, Africa and Haiti.

### Identifying genomic structuring by random forest

Metadata describing the three niche dimensions were collected for all *V. cholerae* strains (Additional file 1). This data includes the location (space) and year (time) of sampling, as well as information about the clinical or environmental source of the strain (habitat). To obtain consistent genotypic annotations, all 274 *V. cholerae* genomes were re-annotated as explained in Methods. Annotated genotypic variables making up the genomic landscape of *V. cholerae* included the presence of protein families identified with CD-HIT [19], genome-wide SNPs [3], functions and subsystems annotated by RAST (Rapid Annotations using Subsystems Technology [20]) and phages identified by using PhiSpy [21] and homology searches (see Methods). Monotonous variables were removed and variables with redundant, highly correlating profiles were merged, yielding a total of 25,305 informative, non-redundant genotypic variables. Where possible, variables were assigned to the SEED level-1 subsystems [9], providing consistent, low-level annotations (see Table 1 and Additional file 3 and Additional file 4).

**Table 1:** **Summary of the genotypic variables**

Attribute	Explanation	Before	After	Annotated
Protein families	CD-HIT clusters [22]	21,146	17,560	9,819
Functions	Level-3 subsystems [20]	4,260	3,105	1,828
SNPs	Marker SNPs [3]	7,880	2,545	659
Subsystems	Level-3 subsystems [20]	706	444	398
Phages	Phages [21]	6	4	4
Clusters	Remove redundancy [6]	0	1,647	0
Total		33,998	25,305	12,708

Number of variables is shown before and after the clustering procedure to remove redundancy [6], as well as the number of variables annotated with level-1 subsystems [9]. The full matrix of 25,305 variables used in the manuscript is provided in Additional file 3 and Additional file 4. See text for details.

We use this wide range of variables to classify the genomes by their niche, in each of the three niche dimensions of space, time, and habitat. We use the RF method, an advanced machine learning approach that uses many decision trees in parallel [7]. For space and habitat dimensions, we applied classification of the strains into six continents and two habitats, respectively. For the time dimension, we applied regression of all genotypic variables against the year of origin. All RFs contained 10,000 decision trees.

In general, all RFs have high prediction accuracies, indicating that the annotated genotypic variables contain enough structure in each of the niche dimensions to allow for classification. The habitat-RFs classified the genomes as clinical or environmental with 89.2% accuracy. The space-RFs classified the genomes by continent with an average of 45.3% accuracy, which is high compared to an expected 16.7% accuracy if the genomes were randomly assigned to one of the six continents. The time-RF explained 62.0% of variation.

**Figure 4:**
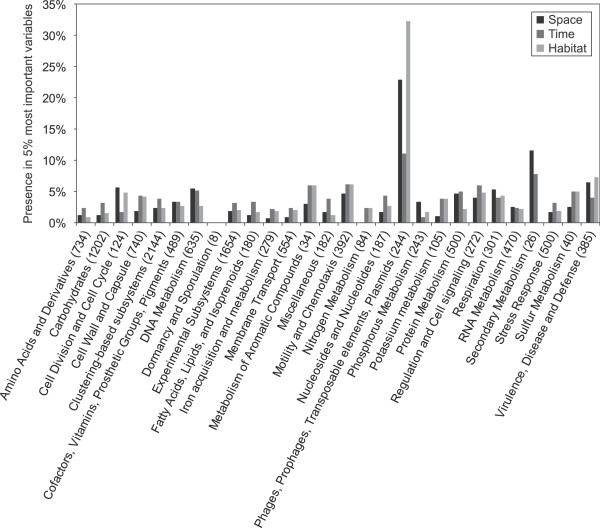
**The important subsystems for each niche dimension.** Presence of level-1 subsystem categories in the top 5% most important functionally annotated genotypic variables for RFs in three niche dimensions. See Additional file 5 for details, the percentage can be changed in that file to dynamically update the bar chart.

Next, the RFs identified which genotypic variables were most important for classification in each of the three niche dimensions explored (Additional file 5). To summarize these results, we analyzed how many of the genotypic variables in each of the SEED level-1 subsystems were present in the top 5% most important variables for each RF. As shown in Figure 4, "Phages, prophages, transposable elements, and plasmids" was the major functional class structuring the genomes in every niche dimension. Of a total of 244 genotypic variables with this level-1 subsystem annotation, 56 (23.0%), 27 (11.1%), and 79 (32.4%) were present among the 1,265 (5% of 25,305) most important variables for space, time, and habitat, respectively. This illustrates the importance of phages and mobile elements for genome evolution, allowing *V. cholerae* to persist in a large volume of the space defined by these three major niche dimensions.

## Conclusions

Here, we investigate how the versatile phenotype of *V. cholerae*, a bacterium that persists across the three major niche dimensions space, time, and habitat, is reflected in its genome. We take a data-driven approach for comparative functional genomics [5] that includes automated annotation of genotypic variables in 28 low-level functional categories [9] and RF machine learning for prioritizing the variables [6]. This approach allows us to fully exploit the genotypic information in many complete genomes simultaneously, and identify which genotypic variables structure the strains in niche space. In each of the three niche dimensions, we find that variations in phages, prophages, transposable elements, and plasmids underlie the diversity and explain most of the structure in the data. These results confirm previous investigations of the separate niche dimensions, showing the importance of these mobile functions in shaping the *V. cholerae* genome. In the spatial dimension, they drive geographic endemism [1, 2]; in time they structure temporal epidemics [3, 23]; and habitat preference is determined by the presence of phage-encoded pathogenicity factors [4].

High-volume genome sequencing is becoming more affordable, and high-throughput pipelines like those employed here are capable of rapidly annotating thousands of genomic elements including SNPs, protein families, functions, and prophages. Exploiting these developments, novel statistical and machine learning analyses can be used to identify connections between genotype and phenotype. Whereas here we apply our approach to niche annotations which are widely available for sequenced strains, it can similarly be used for matching any phenotypic measurement to genotypes (for examples see [5]), and it will only gain in statistical power by including more genomes.

## Methods

### DNA isolation and genome sequencing

Total genomic DNA was isolated using a Wizard Genomic DNA Purification Kit (Promega). Fragment libraries were prepared using the Ion Fragment Library Kit (Life Technologies) with some modifications. First, when necessary the starting material was reduced to 200 ng of genomic DNA (the standard protocol requires 5 μg). Second, we used multiplexed sequencing using molecular barcode adaptors. Genomes were sequenced using Ion Torrent semiconductor sequencing [8] following standard operating procedures for the Personal Genome Machine (PGM).

Sequencing occurred over the course of 2011, the first year that this platform was introduced. To test the applicability of the Ion Torrent system for *de novo* genome sequencing, we included one mate pair library for *V. cholerae* R-18308 that was constructed by substituting Ion adaptors into the prescribed workflow for the SOLiD sequencing system (SOLiD TM 4 System Library Preparation Guide; Life Technologies). The emulsion polymerase chain reaction (PCR) conditions were modified as shown in Table 2 to accommodate a longer template length for the mate pair library. All four nucleotides are introduced in step-wise fashion using the following flow order sequence: TACG TACG TCTG AGCA TCGA TCGA TGTA CAGC. Sixty-five flow order cycles were used to sequence the fragment libraries and 120 flow order cycles were used to accommodate the longer mate pair libraries.

**Table 2:** **Modified emulsion PCR conditions for mate pair library**

Stage	Step	Temperature	Time
Hold	Denature	94°C	6 minutes
Cycle (40)	Denature	94°C	30 seconds
	Anneal	58°C	30 seconds
	Extend	72°C	90 seconds
Cycle (20)	Denature	94°C	30 seconds
	Extend	58°C	18 minutes
Hold		4°C	infinity

The emulsion PCR conditions of the mate pair library construction for Ion Torrent sequencing of *V. cholerae* strain R-18308.

### Assembly, scaffolding, and RAST annotation

Ion Torrent sequencing reads obtained in this study were first cleaned of the internal adaptor (IA) tag using TagCleaner [24] (CTGAGACT allowing up to 1 mismatch occurring within 31 nucleotides (nt) of the 3'-end). Short (<90 nt) reads and long (>200 nt) reads, as well as regions with a quality score <10, were deleted. Reads were then assembled using gsAssembler [25] (version 2.6, default parameters with -notrim, or default options for the mate pair assembly). The recently published *V. cholerae* Amazonia strain R-18332 genome [12] was assembled in the same way. The 203 [3, 10, 11] strains sequenced with an Illumina platform were assembled by using Velvet [26] with a hash length of 31 nt and short paired read types. Assembly and scaffolding statistics are presented in Additional file 2.

For each set of assembled contigs, the most suitable reference genome was selected from the five completely closed *V. cholerae* genomes (2 chromosomes; N16961, O395, MJ-1236, M66-2 and LMA3984-4). To choose the reference, all the contigs were queried against those genomes using blastn (BLAST 2.2.25+ [27]), and the genome with the highest bitscore hit was selected. The contigs were then scaffolded against this reference using *scaffold_builder* 1.0 [28]. The scaffolded contigs were uploaded to RAST [20] for automated gene calling (including frameshift correction) and annotation. Annotation statistics for all strains are presented in Additional file 2.

To create the Circos plot [14], the raw reads from the 38 other genomes sequenced here were mapped to the genome of R-18308 using blastn (BLAST 2.2.25+ [27]), requiring ≥80% nucleotide identity over ≥100 nt for mapping.

### Genome coverage

Sequence coverage of the genomes was estimated by locating orthologous regions that we expected to be present in all the *V. cholerae* genomes. These regions were identified independently of gene annotations, as follows. First, we used progressiveMauve [29] (default parameters) to construct a multiple genome alignment of the 31 *V. cholerae* genomes obtained from the SEED database (Additional file 1). We then extracted 886 local multiple alignment blocks containing all 31 genomes (length between 19 nt and 46,404 nt, average 2,688 nt) and constructed hidden Markov models (HMMs) for each region using hmmbuild (HMMER 3.0 [30], default parameters). These HMMs represent orthologous regions that are present in all *V. cholerae* genomes, whose combined length of 2,381,489 nt covers ~60% of the input genomes. Finally, we queried all 210 genomes and scaffolds with these HMMs using hmmsearch (HMMER 3.0 [30], default parameters) and composed multiple alignments from the hits. We included the nucleotide from the highest scoring hit domain at every position of the HMM alignment and added gaps in regions lacking hits. These orthologous genome regions were used to estimate the sequencing coverage of each genome by calculating the fraction of all the sequence alignments that did not contain gaps.

### Genome-wide marker SNPs

Mutreja et al. previously identified 1,970 reliable marker SNPs in the *V. cholerae* genome [3]. These SNPs are in regions of the genome that are unlikely to have been the subject of horizontal gene transfer, and can be used as reliable genomic markers for the seventh pandemic strains. The 1,970 SNP positions in the corrected version of the N16961 genome (kindly provided by Ankur Mutreja) were mapped to the 274 genomes analyzed in this study by using blastn 2.2.25+ [27] with default parameters. Functions were annotated to SNPs that fell within a gene on the N16961 genome (Additional file 3). We inserted a gap position if a genome did not produce a blastn hit for that SNP. The same procedure was used to obtain the corresponding SNP positions in the four *V. mimicus* genomes (MB-451, VM223, VM573 and VM603), a species closely related to *V. cholerae*, that we included as an outgroup. The full list of SNPs and their variants in all the genomes is provided in Additional file 3. We created a tree from the alignment of high-resolution marker SNPs identified in the 210 *V. cholerae* genomes and four *V. mimicus* genomes (Figure 3), using PhyML 3.0 [31] (NNI tree topology search, initial BioNJ tree, HKY85 model of nucleotide substitutions, 4 discrete categories in the gamma model with an estimated shape parameter (0.680), estimated ts/tv ratio (3.879) and 100 bootstrap iterations).

### Protein families, functions, and subsystems

We constructed protein families using CD-HIT 4.5.7 [19] with a 0.85 sequence identity threshold and a word length of 5. Families that were present in less than five genomes were excluded, yielding a total of 21,146 protein families, which were present in an average of 47.2 genomes each. Because CD-HIT greedily adds proteins to a family, this approach groups protein fragments together that might have been called separately because of frame shift errors that were missed in the automated annotation. The number of 21,146 CD-HIT clusters provides a rough estimate of the V. cholerae pan-genome. However, it should be noted that CD-HIT is not a sophisticated orthology algorithm, so we expect that this number over-estimates the size of the pan-genome, and several of the clusters should actually be merged into larger protein families. Where possible, the clusters were annotated functionally by taking the function(s) that were most often annotated to the proteins in the cluster. The full list is presented in Additional file 3.

In addition to these homology-based protein families, we scored the presence of 4,260 functions and 706 subsystems in the *V. cholerae* genomes using the subsystems annotated by RAST [20]. The full list is presented in Additional file 3.

### Prophages

Phages are important horizontal gene transfer agents that are capable of conveying genetic material between strains that occupy the same niche. We therefore included complete prophages in the *V. cholerae* genomes as genomic variables. Because of their repetitive nature, prophages are one of the principal reasons that contigs cannot be combined during genome assembly. This obstacle is exemplified by the two prophages RS phage (Repeat Sequence, also known as RS1 [32]) and CTX (cholera toxin) in *V. cholerae* El Tor genomes. These two prophages share a stretch of 2,732 nucleotides with 100% identity. The RS prophage is not much longer than that length; it contains an additional 362 nt that differ from the CTX prophage, while the latter has an additional 4,548 nt that are not found in the RS prophage. Thus, the long repeat sequence makes it difficult to accurately assemble and identify these prophages. Moreover, prophages have a high recombination rate, which leads to a mosaic structure. For example, the Kappa prophage varies in length from 32,970 to 35,021 nt, and the Mu-like phage varies in length from 33,001 to 34,078 nt, depending on the host genome.

First, we identified prophages in the database of *V. cholerae* genomes by using PhiSpy [21] with default parameters and the *V. cholerae* N16961 training set. This approach identified eight prophages (Table 3): RS, CTX, Kappa, Mu-like, VP882-like, and WASA. For the RS and CTX phages, we separately retained the common and unique sequences (see above) and called each phage as present only if we identified sequence similarity to this unique region. Second, we used blastn 2.2.25+ [27] with an E-value ≤10^-5^ to map all the genomes to these phage regions, calling them as present if the coverage along the phage sequence exceeded the estimated genome coverage (based on the coverage of orthologous genome regions, see above) and subtracting 5% to account for potential sequencing biases. The resulting presence/absence of each of the phages across all the genomes is presented in Additional file 3.

**Table 3:** **Phages present in the set of 31** ***V. cholerae*** **genomes from the SEED database**

Phage	Source	Initially identified in genome	Genomes
CTX	PhAnToMe id 141904.3, RefSeq id NC_015209	N16961	195
RS	PhAnToMe id 141904.3, RefSeq id NC_015209	N16961	115
Kappa	PhAnToMe id 493906.2; RefSeq id NC_010275	1587, AM-19226, B33, MJ-1236, MO10, NCTC-8457, RC9, RC27	53
WASA	Ref. [33]	INDRE-91/1	18
Mu-like	Identified using PhiSpy [21]	12129(1), TM-11079-80, TMA-21, V51	10
VP882	Identified using PhiSpy [21]	AM-19226, TM-11079-80	5

The phages in this table were mapped by homology to all *V. cholerae* genomes (see Methods).

### Random forest

Random Forest (RF) is a machine learning approach to classifying data that was developed by Breiman and Cutler [7]. The approach is to build a forest of decision trees, each built using a different subset of the data for training. The accuracy of each decision tree is measured using the remaining testing data (cross-validation). The importance of each variable can then be calculated as the average decrease in GINI purity [7] of the trees that results from removing or randomizing the variable. Developed in 2001, this classification approach is becoming more popular in 'omics research [6, 34] because it can incorporate large and noisy datasets with many features, prioritizing the ones that are most important for a certain prediction. Besides the high prediction accuracy, a unique advantage of RF compared to other supervised machine learning techniques (such as support vector machines, SVM) is that many variables can be compared to relatively few classes (the niche dimensions) without the risk of over-training [6]. The technique has been applied to classify proteomics data [35], genome-scale responses to extracellular stimuli [36], microarray data [37], interactome analyses [38] and genome-wide association studies [39].

Here, we use RFs to compare genomic attributes across multiple niche dimensions from hundreds of genomes to reveal which response variables (genotypes) are the most important for separating the observations (genomes) in each dimension. We assembled a matrix of classes (niches or phenotypes) containing the niche metadata for all 274 isolates (Additional file 1), including sampling date (year, time), sampling location (continent, space), and source (clinical or environmental, space). Cases that could not be retrieved were labeled “unknown” and were excluded from the corresponding RFs. We also assembled a matrix of genotypic variables and their presence or absence in every genome (see Table 1). Monotonous variables with identical values in all, or all-but-one of the draft genomes (e.g. universal housekeeping genes) were removed as they would not provide any information about genotypic clustering [6]. Variables with redundant, highly correlating profiles were merged and added as clustered genotypic variables to the matrix (Additional file 6 and Additional file 7). This yielded a total of 25,305 informative, non-redundant genotypic variables. Where, possible, all annotated genotypes including SNPs, protein families, functions, and prophages were assigned to the SEED level-1 subsystems [9], providing consistent, low-level annotations in 28 categories (Table 1 and Additional file 3).

For the space and habitat dimensions, we used classification RFs of the strains into six continents (Africa, Asia, Australia, Europe, North America, and South America) and two habitats (clinical or environmental), respectively. Class imbalance was prevented by jackknife subsampling of genomes from the more abundant classes to a maximum of two times the size of the smallest class [6]. Results are the average of 100 jackknife iterations. As the sampling year is a continuous variable, the RF was trained using regression to fit the data rather than using the classification approach as for the other two niche dimensions. All RFs consisted of 10,000 trees each (the importance scores are robust with this number of trees [40]) and were calculated using the randomForest package 4.5-34 [41] (default parameters) in R version 2.11.0 (The R Project for Statistical Computing; http://www.r-project.org). The importance scores of every genomic variable in each of the RFs are shown in Additional file 5.

All the genomic variables were ranked by the importance score obtained from the RF analysis (Additional file 5). For those variables with a level-1 subsystem annotation (including CD-HIT clusters, functions, subsystems, and SNPs falling within an annotated gene) the fraction of the variables in each of the level-1 subsystem classes found among the top ranked variables was counted. Figure 4 shows the percentage of subsystems in each class found at 5% of the ranks (1,265/25,305 most important variables) for each of the three predictors (time, space, and habitat).

### Availability of supporting data

The data sets supporting the results of this article are available in the Sequence Read Archive (SRA), http://www.ebi.ac.uk/ena/data/view/ERP001410 and http://www.ebi.ac.uk/ena/data/view/ERP001412.

## Electronic supplementary material

Additional file 1: **List of** ***V. cholerae*** **strains.** *V. cholerae* strains included in this study. Strain name, source of the sequence data, identifier, year of isolation, country and continent of origin, and habitat are indicated. (XLSX 20 KB)

Additional file 2: **Assembly statistics.** Assembly statistics of *V. cholerae* genome sequences. Strain name, genome identifier, source, number of reads, average read length, number of nucleotides sequenced, number of contigs, contig N50 length, length of the longest contig, SEED identifier of the scaffolding reference used by *scaffold_builder*, number of scaffolds, scaffold N50 length, length of the longest scaffold, total genome length, estimated coverage, and sequencing depth are indicated. (XLSX 50 KB)

Additional file 3: ***V. cholerae*** **protein families.** This table contains one class of genomic variables, the CD-HIT protein families and their presence in all *V. cholerae* genomes. Variable identifier, type of variable (CD-HIT), function, and SEED level-1 subsystem are indicated. (XLSX 14 MB)

Additional file 4: **Genomic variables other than protein families.** Other genomic variables present in all *V. cholerae* genomes. Variable identifier, type of variable (highly correlating cluster; RAST function; prophage; marker SNP; SEED subsystem), function, and SEED level-1 subsystem are indicated. (XLSX 6 MB)

Additional file 5: **Importance of variables by subsystem.** Summary of the SEED level-1 subsystems for the prediction of each of the niche dimensions. Average importance scores and ranks of all variables for each RF (space, time, and habitat) are indicated in the subsequent tabs. (XLSX 4 MB)

Additional file 6: **Diagram of merged clusters of redundant variables.** Cytoscape [42] representation of clusters of variables with highly correlating profiles (Pearson r >0.98 and Spearman r >0.95). These clusters were merged to avoid redundancy in the RF analysis. See Additional file 7 for the complete list of merged variables. (PDF 1 MB)

Additional file 7: **List of merged clusters of redundant variables.** Variables with highly correlating profiles (Pearson r >0.98 and Spearman r >0.95) were merged into clusters to avoid redundancy in the RF analysis. Cluster identifier, number of merged variables, and a list of merged variables are indicated. (XLSX 80 KB)

## Abbreviations

CTXφ: Cholera toxin phage
GWAS-M: Genome-wide association studies for microbes
HMM: Hidden Markov model
IA: Internal adaptor
PGM: Ion torrent personal genome machine
RF: Random forest
SNP: Single-nucleotide polymorphism
SVM: Support vector machine
TCP: Toxin co-regulated pilus
WASA: West Africa-South America phage.

## References

[1] Boucher Y, Cordero OX, Takemura A, Hunt DE, Schliep K, Bapteste E, Lopez P, Tarr CL, Polz MF (2011). Local mobile gene pools rapidly cross species boundaries to create endemicity within global vibrio cholerae populations. MBio.

[2] Shapiro BJ, Friedman J, Cordero OX, Preheim SP, Timberlake SC, Szabo G, Polz MF, Alm EJ (2012). Population genomics of early events in the ecological differentiation of bacteria. Science.

[3] Mutreja A, Kim DW, Thomson NR, Connor TR, Lee JH, Kariuki S, Croucher NJ, Choi SY, Harris SR, Lebens M, Niyogi SK, Kim EJ, Ramamurthy T, Chun J, Wood JL, Clemens JD, Czerkinsky C, Nair GB, Holmgren J, Parkhill J, Dougan G (2011). Evidence for several waves of global transmission in the seventh cholera pandemic. Nature.

[4] Waldor MK, Mekalanos JJ (1996). Lysogenic conversion by a filamentous phage encoding cholera toxin. Science.

[5] Dutilh BE, Backus L, Edwards RA, Wels M, Bayjanov JR, van Hijum SA (2013). Explaining microbial phenotypes on a genomic scale: GWAS for microbes. Brief Funct Genomics.

[6] Bayjanov JR, Molenaar D, Tzeneva V, Siezen RJ, van Hijum SA (2012). PhenoLink–a web-tool for linking phenotype to ~ omics data for bacteria: application to gene-trait matching for Lactobacillus plantarum strains. BMC Genomics.

[7] Breiman L (2001). Random forests. Mach Learn.

[8] Rothberg JM, Hinz W, Rearick TM, Schultz J, Mileski W, Davey M, Leamon JH, Johnson K, Milgrew MJ, Edwards M, Hoon J, Simons JF, Marran D, Myers JW, Davidson JF, Branting A, Nobile JR, Puc BP, Light D, Clark TA, Huber M, Branciforte JT, Stoner IB, Cawley SE, Lyons M, Fu Y, Homer N, Sedova M, Miao X, Reed B (2011). An integrated semiconductor device enabling non-optical genome sequencing. Nature.

[9] Overbeek R, Begley T, Butler RM, Choudhuri JV, Chuang HY, Cohoon M, de Crecy-Lagard V, Diaz N, Disz T, Edwards R, Fonstein M, Frank ED, Gerdes S, Glass EM, Goesmann A, Hanson A, Iwata-Reuyl D, Jensen R, Jamshidi N, Krause L, Kubal M, Larsen N, Linke B, McHardy AC, Meyer F, Neuweger H, Olsen G, Olson R, Osterman A, Portnoy V (2005). The subsystems approach to genome annotation and its use in the project to annotate 1000 genomes. Nucleic Acids Res.

[10] Hasan NA, Choi SY, Eppinger M, Clark PW, Chen A, Alam M, Haley BJ, Taviani E, Hine E, Su Q, Tallon LJ, Prosper JB, Furth K, Hoq MM, Li H, Fraser-Liggett CM, Cravioto A, Huq A, Ravel J, Cebula TA, Colwell RR (2012). Genomic diversity of 2010 Haitian cholera outbreak strains. Proc Natl Acad Sci U S A.

[11] Hendriksen RS, Price LB, Schupp JM, Gillece JD, Kaas RS, Engelthaler DM, Bortolaia V, Pearson T, Waters AE, Upadhyay BP, Shrestha SD, Adhikari S, Shakya G, Keim PS, Aarestrup FM (2011). Population genetics of Vibrio cholerae from Nepal in 2010: evidence on the origin of the Haitian outbreak. MBio.

[12] Thompson CC, Marin MA, Dias GM, Dutilh BE, Edwards RA, Iida T, Thompson FL, Vicente AC (2011). Genome sequence of the human pathogen Vibrio cholerae Amazonia. J Bacteriol.

[13] Chain PS, Grafham DV, Fulton RS, Fitzgerald MG, Hostetler J, Muzny D, Ali J, Birren B, Bruce DC, Buhay C, Cole JR, Ding Y, Dugan S, Field D, Garrity GM, Gibbs R, Graves T, Han CS, Harrison SH, Highlander S, Hugenholtz P, Khouri HM, Kodira CD, Kolker E, Kyrpides NC, Lang D, Lapidus A, Malfatti SA, Markowitz V, Metha T (2009). Genomics. Genome project standards in a new era of sequencing. Science.

[14] Krzywinski M, Schein J, Birol I, Connors J, Gascoyne R, Horsman D, Jones SJ, Marra MA (2009). Circos: an information aesthetic for comparative genomics. Genome Res.

[15] Popovic T, Fields PI, Olsvik O, Wells JG, Evins GM, Cameron DN, Farmer JJ, Bopp CA, Wachsmuth K, Sack RB, Albert MJ, Nair GB, Shimada T, Feeley JC (1995). Molecular subtyping of toxigenic Vibrio cholerae O139 causing epidemic cholera in India and Bangladesh, 1992-1993. J Infect Dis.

[16] Waldor MK, Colwell R, Mekalanos JJ (1994). The Vibrio cholerae O139 serogroup antigen includes an O-antigen capsule and lipopolysaccharide virulence determinants. Proc Natl Acad Sci U S A.

[17] Johnson JA, Salles CA, Panigrahi P, Albert MJ, Wright AC, Johnson RJ, Morris JG (1994). Vibrio cholerae O139 synonym bengal is closely related to Vibrio cholerae El Tor but has important differences. Infect Immun.

[18] Reimer AR, Van Domselaar G, Stroika S, Walker M, Kent H, Tarr C, Talkington D, Rowe L, Olsen-Rasmussen M, Frace M, Sammons S, Dahourou GA, Boncy J, Smith AM, Mabon P, Petkau A, Graham M, Gilmour MW, Gerner-Smidt P (2011). Comparative genomics of Vibrio cholerae from Haiti, Asia, and Africa. Emerg Infect Dis.

[19] Huang Y, Niu B, Gao Y, Fu L, Li W (2010). CD-HIT Suite: a web server for clustering and comparing biological sequences. Bioinformatics.

[20] Aziz RK, Bartels D, Best AA, DeJongh M, Disz T, Edwards RA, Formsma K, Gerdes S, Glass EM, Kubal M, Meyer F, Olsen GJ, Olson R, Osterman AL, Overbeek RA, McNeil LK, Paarmann D, Paczian T, Parrello B, Pusch GD, Reich C, Stevens R, Vassieva O, Vonstein V, Wilke A, Zagnitko O (2008). The RAST Server: rapid annotations using subsystems technology. BMC Genomics.

[21] Akhter S, Aziz RK, Edwards RA (2012). PhiSpy: a novel algorithm for finding prophages in bacterial genomes that combines similarity- and composition-based strategies. Nucleic Acids Res.

[22] Li W, Jaroszewski L, Godzik A (2001). Clustering of highly homologous sequences to reduce the size of large protein databases. Bioinformatics.

[23] Garza DR, Thompson CC, Loureiro EC, Dutilh BE, Inada DT, Junior EC, Cardoso JF, Nunes MR, de Lima CP, Silvestre RV, Nunes KN, Santos EC, Edwards RA, Vicente AC, de Sa Morais LL (2012). Genome-wide study of the defective sucrose fermenter strain of Vibrio cholerae from the Latin American cholera epidemic. PLoS One.

[24] Schmieder R, Lim YW, Rohwer F, Edwards R (2010). TagCleaner: identification and removal of tag sequences from genomic and metagenomic datasets. BMC Bioinformatics.

[25] Margulies M, Egholm M, Altman WE, Attiya S, Bader JS, Bemben LA, Berka J, Braverman MS, Chen YJ, Chen Z, Dewell SB, Du L, Fierro JM, Gomes XV, Godwin BC, He W, Helgesen S, Ho CH, Irzyk GP, Jando SC, Alenquer ML, Jarvie TP, Jirage KB, Kim JB, Knight JR, Lanza JR, Leamon JH, Lefkowitz SM, Lei M, Li J (2005). Genome sequencing in microfabricated high-density picolitre reactors. Nature.

[26] Zerbino DR, Birney E (2008). Velvet: algorithms for de novo short read assembly using de Bruijn graphs. Genome Res.

[27] Camacho C, Coulouris G, Avagyan V, Ma N, Papadopoulos J, Bealer K, Madden TL (2009). BLAST+: architecture and applications. BMC Bioinformatics.

[28] Silva GGZ, Dutilh BE, Matthews TD, Elkins K, Schmieder R, Dinsdale EA, Edwards RA (2013). Combining de novo and reference-guided assembly with scaffold_builder. Source Code Biol Med.

[29] Darling AE, Mau B (2010). Perna NT: progressiveMauve: multiple genome alignment with gene gain, loss and rearrangement. PLoS One.

[30] Eddy SR (2009). A new generation of homology search tools based on probabilistic inference. Genome Inform.

[31] Guindon S, Gascuel O (2003). A simple, fast, and accurate algorithm to estimate large phylogenies by maximum likelihood. Syst Biol.

[32] Hassan F, Kamruzzaman M, Mekalanos JJ, Faruque SM (2010). Satellite phage TLCphi enables toxigenic conversion by CTX phage through dif site alteration. Nature.

[33] Morais LL, Garza DR, Loureiro EC, Nunes KN, Vellasco RS, da Silva CP, Nunes MR, Thompson CC, Vicente AC, Santos EC (2012). Complete genome sequence of a sucrose-nonfermenting epidemic strain of vibrio cholerae O1 from Brazil. J Bacteriol.

[34] Touw WG, Bayjanov JR, Overmars L, Backus L, Boekhorst J, Wels M, van Hijum SA (2012). Data mining in the Life Sciences with Random Forest: a walk in the park or lost in the jungle?. Brief Bioinform.

[35] Barrett JH, Cairns DA (2008). Application of the random forest classification method to peaks detected from mass spectrometric proteomic profiles of cancer patients and controls. Stat Appl Genet Mol Biol.

[36] Bienkowska JR, Dalgin GS, Batliwalla F, Allaire N, Roubenoff R, Gregersen PK, Carulli JP (2009). Convergent Random Forest predictor: methodology for predicting drug response from genome-scale data applied to anti-TNF response. Genomics.

[37] Stiglic G, Rodriguez JJ, Kokol P (2011). Rotation of random forests for genomic and proteomic classification problems. Adv Exp Med Biol.

[38] Ananthasubramanian S, Metri R, Khetan A, Gupta A, Handen A, Chandra N, Ganapathiraju M (2012). Mycobacterium tuberculosis and Clostridium difficille interactomes: demonstration of rapid development of computational system for bacterial interactome prediction. Microb Inform Exp.

[39] Chung RH, Chen YE (2012). A two-stage random forest-based pathway analysis method. PloS one.

[40] Calle ML, Urrea V (2011). Letter to the editor: Stability of Random Forest importance measures. Brief Bioinform.

[41] Liaw A, Wiener M (2002). Classification and regression by random Forest. R News.

[42] Smoot ME, Ono K, Ruscheinski J, Wang PL, Ideker T (2011). Cytoscape 2.8: new features for data integration and network visualization. Bioinformatics.

