# Supplementary figures and images for: Comparative genomics of 274 Vibrio cholerae genomes reveals mobile functions structuring three niche dimensions
Source: BMC Genomics. 2014 Aug 5;15(1):654. doi: 10.1186/1471-2164-15-654 (PMC4141962; doi:10.1186/1471-2164-15-654)

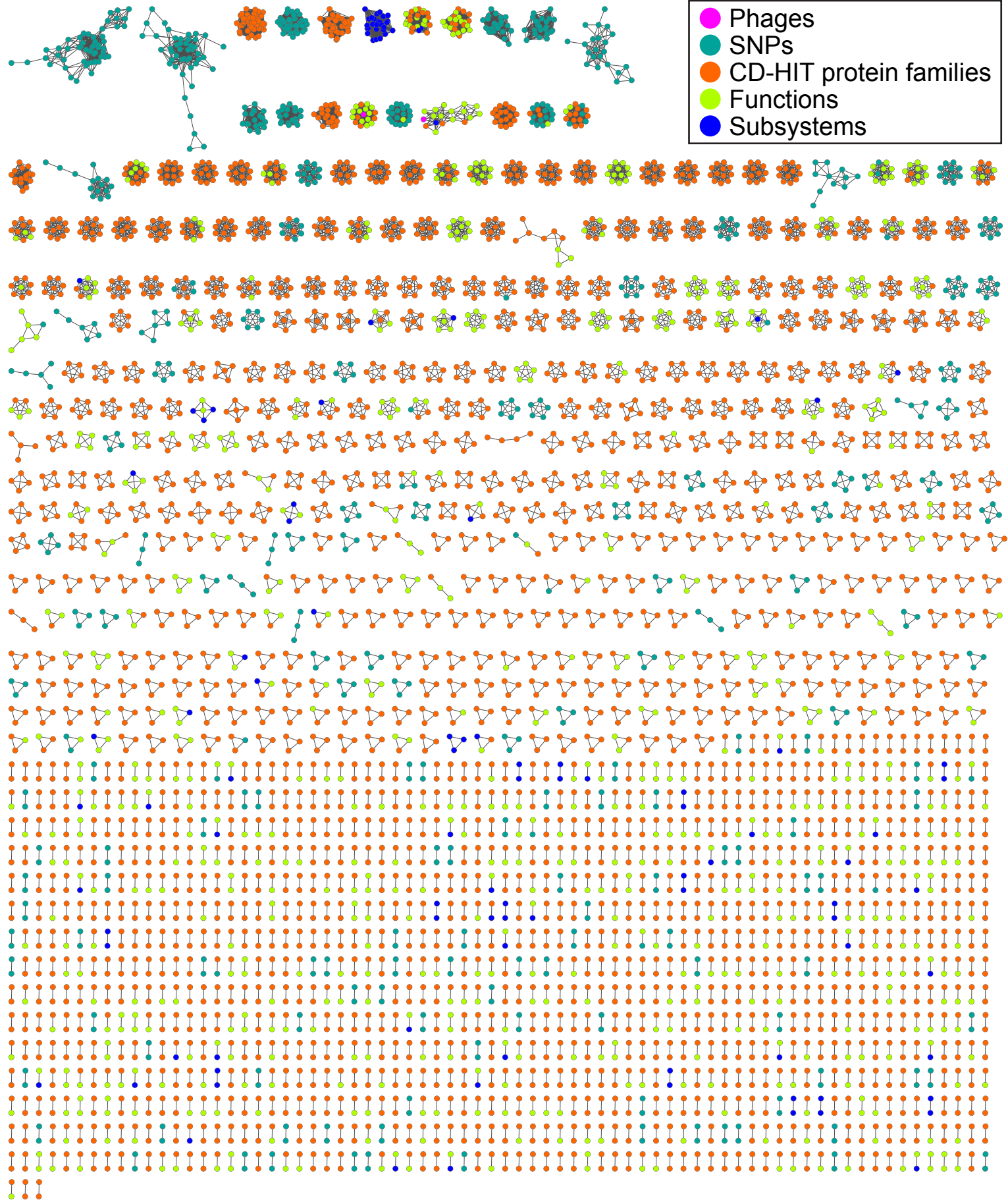

Supplement: Supplementary file 6 — Additional file 6: Diagram of merged clusters of redundant variables. Cytoscape [42] representation of clusters of variables with highly correlating profiles (Pearson r >0.98 and Spearman r >0.95). These clusters were merged to avoid redundancy in the RF analysis. See Additional file 7 for the complete list of merged variables. (PDF 1 MB) [file 12864_2014_6365_MOESM6_ESM.pdf]
